# Efficient microresonator frequency combs

**DOI:** 10.1186/s43593-024-00075-5

**Published:** 2024-10-10

**Authors:** Qi-Fan Yang, Yaowen Hu, Victor Torres-Company, Kerry Vahala

**Affiliations:** 1 State Key Laboratory for Artificial Microstructure and Mesoscopic Physics and Frontiers Science Center for Nano-optoelectronics, School of Physics, Peking University, Beijing, China; 2 John Paulson School of Engineering and applied science, Harvard University, Cambridge, Boston, USA; 3 Department of Microtechnology and Nanoscience, Chalmers University of Technology, Gothenburg, Sweden; 4 T. J. Watson Laboratory of Applied Physics, California Institute of Technology, Pasadena, USA

**Keywords:** Optical frequency comb, Optical microresonator, Nonlinear photonics

## Abstract

The rapid development of optical frequency combs from their table-top origins towards chip-scale platforms has opened up exciting possibilities for comb functionalities outside laboratories. Enhanced nonlinear processes in microresonators have emerged as a mainstream comb-generating mechanism with compelling advantages in size, weight, and power consumption. The established understanding of gain and loss in nonlinear microresonators, along with recently developed ultralow-loss nonlinear photonic circuitry, has boosted the optical energy conversion efficiency of microresonator frequency comb (microcomb) devices from below a few percent to above 50%. This review summarizes the latest advances in novel photonic devices and pumping strategies that contribute to these milestones of microcomb efficiency. The resulting benefits for high-performance integration of comb applications are also discussed before summarizing the remaining challenges.

## Introduction

Optical frequency combs (OFCs) [1, 2] have fundamentally transformed frequency metrology, spectroscopy, timekeeping, and a range of other applications. In the frequency domain, OFCs consist of an array of spectral lines with fixed phase relationships separated by the repetition frequency. When the array has an octave-level frequency span, it is possible to control the absolute frequencies of the comb lines through the f-2f self-referencing technique. This bidirectional linkage results in a coherent unification of optical and radiofrequency electromagnetic waves [3].

Widespread application of OFCs sparked interest in various realizations, including mode-locked lasers and electro-optic (EO) combs [3, 4]. However, the miniaturization of OFCs was hindered by the complexity of the setups involving discrete free-space or fiber-optic components. In 2007, a novel microresonator-based frequency comb, or “microcomb”, emerged as a promising solution for compact OFC creation [5]. These devices utilized cascaded four-wave mixing (FWM) [6, 7] that was resonantly enhanced in high-quality-factor (high-*Q*) whispering gallery resonators [8]. As regenerative devices, they have a threshold pump power that is inversely proportional to $Q^2$ [6], and exhibit pumping threshold powers below 1 mW. Moreover, Kerr-induced FWM is ubiquitous in low optical loss dielectrics, enabling demonstrations of these FWM microcombs across a wide range of platforms [9]. However, generating stable FWM OFCs was challenging due to the complex dynamics associated with their formation [10]. This challenge was overcome by the realization of dissipative Kerr solitons [11, 12], a concept originally developed in fiber-optic resonators [13, 14]. Soliton microcombs have been demonstrated in many materials [11, 15–21], and provide broad spectra that can be orders of magnitude larger than semiconductor mode-locked lasers [22]. The waveforms and spectral envelopes of dissipative Kerr microcombs vary with the group velocity dispersion of the microresonator. Conventional soliton microcombs (sometimes called bright soliton combs) require anomalous dispersion [11], but normal dispersion microcombs (sometimes called dark pulse combs) are also possible [23, 24].

Even more recently, resonant EO microcombs using high-*Q* microresonators fabricated on thin-film lithium niobate on insulator wafers have appeared [25, 26]. Bulk EO combs [27] generate the comb spectra through direct EO modulation of an optical carrier wave. They are naturally coherent with line spacing determined by the frequency of the driving microwave. The new integrated lithium niobate waveguides offer low half-wave voltage and high bandwidth for more efficient EO modulation [28], and when integrated into a ring microresonator the effective interaction length for modulation is greatly increased [19, 20, 29]. The optical bandwidth of resonant EO microcombs is determined by the power of the microwave driver, in addition to the *Q* and dispersion of the microresonator. Alternatively, resonant EO microcombs are also possible by placing EO microresonators within a microwave-enhancement cavity [30, 31].

Microcombs exhibit outstanding coherence properties, enabling applications such as spectroscopy [32–36], optical frequency synthesis [37], astronomical calibration [38, 39], compact atomic clocks [40], optical communications [41–43], LiDAR [44–48], and microwave photonics [49–54]. Accordingly, the research activities on microcombs have quickly transitioned from device-level demonstrations to system-level integration. Here, thanks to the emergence of ultralow-loss Si$_3$N$_4$ waveguide technologies, microcomb technology has incorporated state-of-the-art integrated photonics [22] to advance integration levels [55–61], paving the way towards mass production of compact OFCs suitable for non-laboratory deployments.

**Fig. 1:**
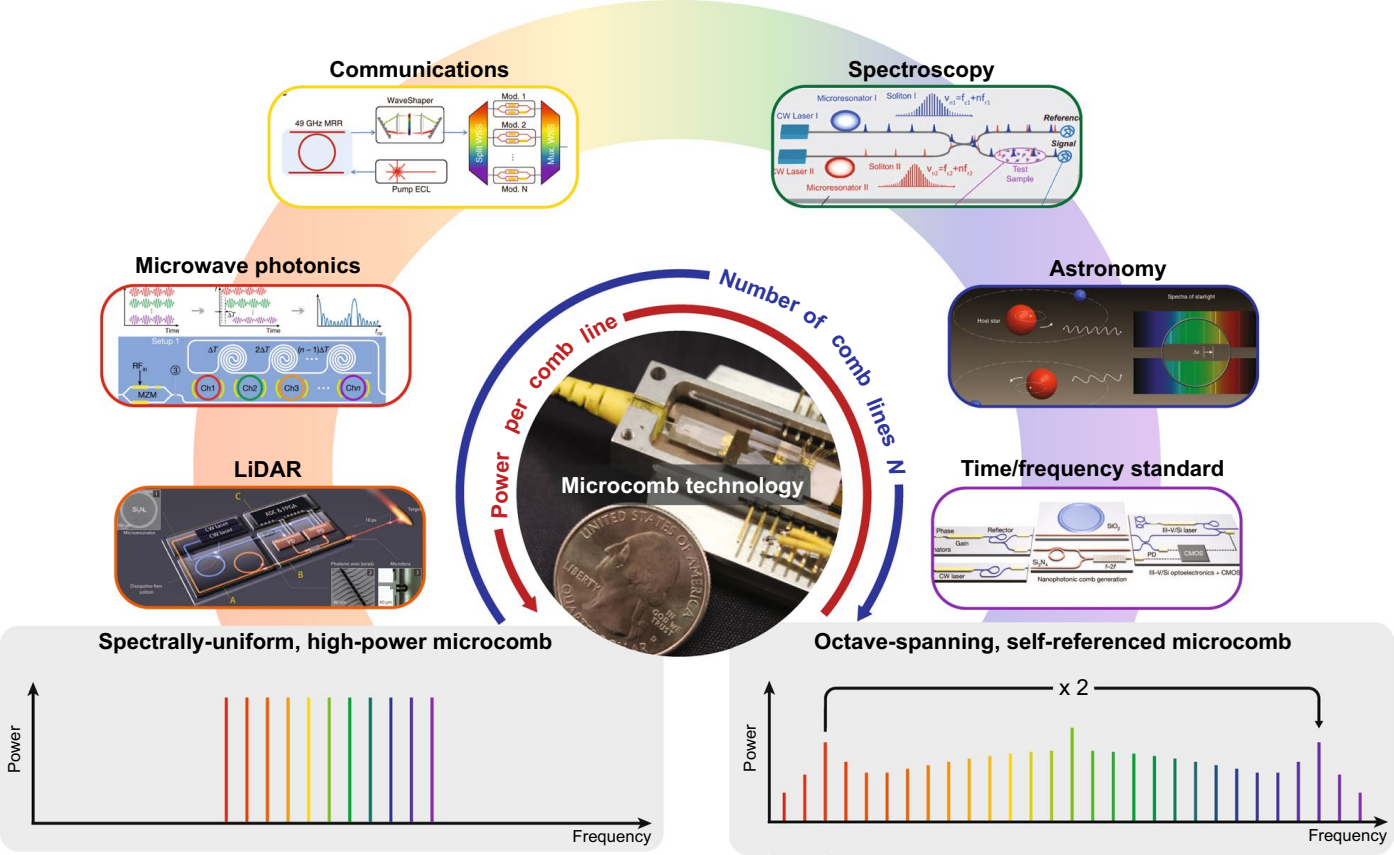
Application-driven development of microcombs. The arrows around the microcomb module [55] indicate the relative importance of the power and number of comb lines for different applications, which include microwave photonics [49], LiDAR [44], communications [41], spectroscopy [32], astronomical calibration [38], and time/frequency standard [37]. Note that the requirements may vary depending on the particular system under consideration. Figures reproduced with permission from: microcomb technology, ref. [55], Copyright 2020 Springer Nature; microwave photonics, ref. [49], Copyright 2022 Springer Nature; LiDAR, ref. [44], Copyright 2018 AAAS; communications [41], ref. [41], Copyright 2020 Springer Nature; spectroscopy, ref. [32], Copyright 2016 AAAS; astronomical calibration, ref. [38], Copyright 2019 Springer Nature; time/frequency standard, ref. [37], Copyright 2018 Springer Nature

A critical metric in system applications of microcombs is their power efficiency, and we review the recent advances in generating high-efficiency microcombs. Typically, efficiency is strongly determined by the number of comb lines, impacting comb bandwidth and comb power per line as required by various applications (Fig. 1). Based on the technology readiness levels and application potential, we focus on two types of microcombs: Kerr microcombs [9, 12] and resonant EO microcombs [19, 20, 30]. We give the major limiting factors of microcomb efficiency and discuss two main strategies to overcome these limitations: using coupled resonators and increasing pumping bandwidth. The latest attempts to create flat-top microcombs are also noted. Finally, we provide a perspective of how high-efficiency microcombs can further advance real-world applications and outline the remaining challenges.

## Principles of microcomb efficiency

**Fig. 2:**
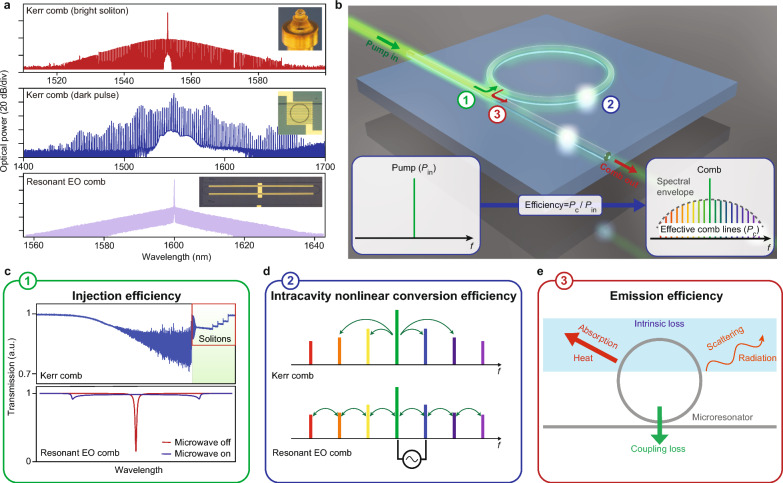
Definition of the microcomb efficiency and its limitations.** a** Representative optical spectra of several types of microcombs [11, 19, 23]. The microresonator devices are also displayed in the insets. **b** Schematic configuration of a typical microcomb. The microcomb efficiency is defined as the ratio between the comb power within the spectral envelope and the power of the input monochromatic pump laser. **c**–**e** Three major limits to the microcomb efficiency, namely the injection efficiency [11, 19], the nonlinear conversion efficiency, and the emission efficiency [82]. Figures reproduced with permission from: **a** Kerr microcomb (bright soliton), ref. [11], Copyright 2014 Springer Nature; **a** Kerr microcomb (dark pulse), ref. [23], Copyright 2015 Springer Nature; **a** Resonant EO microcomb, ref. [19], Copyright 2019 Springer Nature; **c** Kerr microcomb, ref. [11], Copyright 2014 Springer Nature; **c** Resonant EO microcomb, ref. [19], Copyright 2019 Springer Nature; **e** ref. [82], Copyright 2022 Springer Nature

The configuration for generating Kerr and resonant EO microcombs (representative spectra presented in Fig. 2a) has a standard layout that typically involves a continuously pumped, waveguide-coupled microresonator (Fig. 2b). In addition to Kerr effect or EO effect (also known as the Pockels effect), the formation dynamics of microcombs may involve other nonlinear optical processes such as second/half-harmonic generation [18, 62, 63] and stimulated Raman/Brillouin scattering [64–66]. These processes, either independently or collectively, can lead to the formation of equidistant spectral lines within a spectral envelope. However, a significant amount of the pump power may not participate in the comb formation process, resulting in a strong pump line that exceeds the spectral envelope. In practical applications, this residual pump power needs to be attenuated to prevent saturation of the photodetectors. Therefore, in this review, we have adopted the widely accepted definition of “microcomb efficiency” as the ratio of power in the comb lines (excluding any residual pump power that exceeds the comb envelope) to the power of the pump laser [15, 67, 68]. It is worth mentioning that such optical-to-optical conversion efficiency excludes the microwave power consumed in EO microcombs, which can amount to a few Watts. It also does not include the heater power consumption used to tune the resonances thermally. The wall plug efficiency is discussed in the conclusion. Typically, bright soliton Kerr microcombs and resonant EO microcombs exhibit efficiencies of a few percent or less [19, 67]. On the other hand, dark pulse microcombs can achieve efficiencies close to 50% [68]. Detailed analytical expressions for their efficiencies are provided in Box 1. The subsequent section delves into the three key factors that limit the efficiency of microcombs (Fig. 2c-e). While this review focuses on comb efficiency, trade-offs among different metrics, such as efficiencies, comb span, spectral shape, etc, may exist.

**Table:** 

Box 1: Efficiency of typical Kerr microcombs and resonant EO microcombs	
Kerr microcombs: The formation dynamics of Kerr microcombs are governed by the mean-field Lugiato-Lefever equation [11, 69–71]. This equation provides a foundation for understanding the relationship between the efficiency of a single bright soliton and the detuning ($\Delta$) between the pump and the mode. Specifically, it has been shown that the bright soliton efficiency is given by $\Gamma _{{{\text{BS}}}}$ [15, 72, 73]	
$$\Gamma _\text{BS} = \frac{2\eta A_\text{eff}}{n_2 Q P_\text{in}}\sqrt{-2nc\beta _2\Delta }.\qquad \qquad \qquad (1)$$	
Here, $\eta$ represents the loading factor, comparing the coupling loss ($\kappa _\text{e}$) to the total loss ($\kappa$) of the microresonator. Other parameters include the nonlinear effective mode area ($A_\text{eff}$), the linear (*n*) and nonlinear ($n_2$) refractive indices, the quality factor of the microresonator (*Q*), the input pump power ($P_\text{in}$), the speed of light in vacuum (*c*), and the group velocity dispersion of the mode ($\beta _2$).	
To sustain solitons within the microresonator, the detuning must remain below a maximum value proportional to the input pump power. This maximum detuning limits the efficiency of a single bright soliton microcomb [15, 72, 73]. Moreover, recent theoretical and experimental work has extended this understanding by considering the spectral envelope shift due to Raman and dispersive effects [67]. This leads to the following expression for the optimal efficiency:	
$$\Gamma _\text{BS} =2 \pi ^2 \eta ^2 \frac{\tau }{t_\text{R}} \text{sech}^2(\pi \Omega \tau /2) \approx \frac{3.5 \eta ^2}{N} \text{sech}^2(\pi \Omega \tau /2),\qquad \qquad \qquad (2)$$	
where $\tau =\sqrt{-c\beta _2/(2n\Delta )}$ is the pulse duration, $t_\text{R}=1/\text{FSR}$ represents the round-trip time of the microresonator, and $\Omega$ represents the Raman spectral envelope shift. The number of comb lines within the 3 dB bandwidth of the spectral envelope (*N*) is approximately $0.18 t_\text{R}/\tau$ for sech$^2$-type soliton pulses. This scaling underscores the crucial role of temporal overlap between the driving field and the soliton pulse in determining the conversion efficiency, as well as the difficulty to generate a large number of comb lines, especially in Raman-active materials [74]. Indeed, the relatively low efficiencies of bright solitons can be inferred from the low duty cycles ($\tau /t_\text{R}\ll 1$). The coexistence of multiple solitons within a microresonator would lead to a multiplication of efficiency [75–79]. Nevertheless, this coexistence comes with a trade-off: the optical spectra of multiple solitons exhibit interference-induced corrugations, differing from the well-defined $\text{sech}^2$ spectral envelope of a single bright soliton.	
The temporal waveform of dark pulse microcombs typically exhibits a flat-top pulse profile, also known as a “platicon” [24]. These pulses possess a relatively larger duty cycle compared to bright solitons. Despite the absence of analytical solutions for the pulse envelope, studies have revealed an upper limit for the efficiency of dark pulses, given by the equation	
$$\Gamma _\text{DP} = 4\eta ^2 \frac{\tau }{t_\text{R}}\left(1-\frac{\tau }{t_\text{R}}\right),\qquad \qquad \qquad (3)$$	
where $\tau$ denotes the pulse width at full-width-at-half-maximum [80]. The efficiency achieves its peak value ($\eta ^2$) when the duty cycle is 50%.	
Resonant EO microcombs: The efficiency of the resonant EO microcomb can be described by the following equation [20, 81]:	
$$\Gamma _\text{EO}=\eta \left(1-\left|\frac{1-2\eta +\eta _{\text{MW}}}{1+\eta _{\text{MW}}}\right|^2\right),\qquad \qquad \qquad (4)$$	
where $\eta _{\text{MW}}=\kappa _\text{MW}/\kappa$ represents the microwave-induced loading factor, in which $\kappa$ is the linewidth of the resonator and $\kappa _\text{MW}$ is the additional damping on the pump that is induced by the microwave drive. The $\kappa _\text{MW}$ is given by $\kappa _\text{MW}=\kappa [\sqrt{1+\beta ^2/(\kappa ^2 t_\text{R}^2)}-1]$ where $\beta$ is the modulation strength. To generate broadband EO microcomb, the system requires $\beta \gg \pi$. As a result, the microwave-induced loading factor $\eta _\text{MW}$ typically greatly exceed 1 (e.g. $\kappa _\text{MW} \sim 10 \text{GHz}, \kappa \sim 100 \text{MHz}$ on the platform of thin-film lithium niobate [20]). Consequently, the efficiency of the resonant EO microcomb is relatively low ($\sim$0.3%) [19, 30].

*Injection efficiency*. Utilizing the mean-field approximation, the input–output relation of a high-*Q* microresonator can be expressed by the following equation:

5 $$\begin{aligned} \frac{\text{d}a}{\text{d}t} = -(\frac{\kappa }{2} + i\Delta )a + \sqrt{\kappa _\text{e}P_\text{in}}, \end{aligned}$$

where $|a|^2$ represents the intracavity energy, $\kappa$ denotes the total loss rate of the microresonator, $\kappa _\text{e}$ is the waveguide-resonator coupling rate, and $\Delta$ represents the frequency detuning between the mode and the pump. To simplify the analysis, a loading factor $\eta = \kappa _\text{e}/\kappa$ is commonly introduced as a parameter to compare these loss rates. The ideal pumping state for a microresonator is achieved under the condition of “critical coupling” [83], where all pump power is confined and undergoes internal circulation within the microresonator, without being transmitted. For linear microresonators, this condition is met when the pump frequency is precisely tuned to the resonance ($\Delta = 0$) and the loading factor equals 1/2.

However, in resonant EO microcombs, microwave modulation introduces additional losses due to the generation of new frequency components. This results in a reduced effective loading factor [20]. As for Kerr microcombs, apart from Kerr-induced nonlinear losses, the generation of bright solitons requires far red-detuned conditions ($\Delta \gg \kappa$) [11, 12]. Consequently, in both cases, the critical coupling conditions are not satisfied, leading to the majority of pump power being transmitted through the waveguide instead of participating in the comb generation process (Fig. 2c). These reductions in injection efficiency can be dynamically addressed through the general critical coupling approach, which will be discussed in Section 3.

**Fig. 3:**
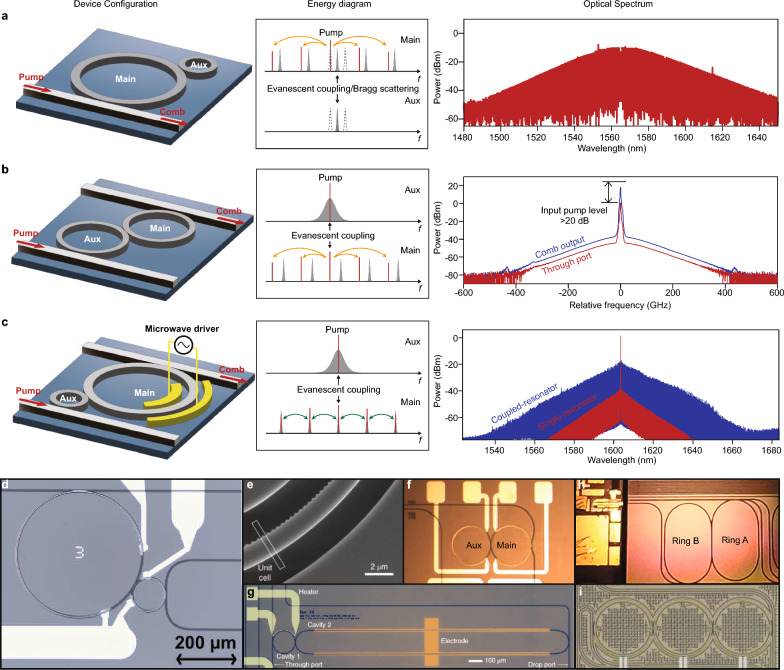
High-efficiency microcombs in coupled-microresonators. **a**–**c** Left panels: configurations of coupled microresonators. Middle panels: energy diagrams of the microresonators. Right panels: representative optical spectra of high-efficiency microcombs. **d**–**i** Demonstrated coupled-resonator devices. Figures reproduced with permission from: **a** optical spectrum, ref. [84], Copyright 2023 Springer Nature; **b** optical spectrum, ref. [85], Copyright 2019 Springer Nature; **c** optical spectrum, ref. [20], Copyright 2022 Springer Nature; **d** ref. [84], Copyright 2023 Springer Nature; **e** ref. [86], Copyright 2021 Springer Nature; **f** ref. [87], Copyright 2019 OSA; **g** ref. [20], Copyright 2022 Springer Nature; **h** ref. [61], Copyright 2023 OSA; **i** ref. [88], Copyright 2022 AAAS

*Intracavity nonlinear conversion efficiency*. The conversion of the intracavity pump laser into comb lines depends on the electrical or optical driving applied to the microresonator (Fig. 2d). In the case of resonant EO microcombs, their intracavity conversion efficiency is determined by the strength of the EO modulation, which is influenced by the microwave power and the half-wave voltage of the modulator [20]. On the other hand, for Kerr microcombs, the conversion relies on the pumping intensity of the microresonator [89]. In both scenarios, the nonlinear conversion efficiency can be enhanced at a given electrical/optical power by utilizing materials with high nonlinearities. Notably, the FWM process in high-*Q* microresonators exhibits remarkable efficiency, as bright solitons emerging from the drop-port of the microresonator have demonstrated that up to 75% of the intracavity pump power can be effectively transformed into comb lines [89].

Moreover, the occurrence of higher-order dispersion or stimulated Raman scattering can shift the carrier frequency of the wavepackets from their pump frequency [16, 18, 67, 90]. This, in turn, can result in an exponential decrease in the conversion efficiency [67]. In strongly Raman-active media, this effect can pose a fundamental limit on the bandwidth, thus prohibiting the generation of broadband microcombs [74].

*Emission efficiency*. The generated microcomb faces several types of dissipation such as scattering and material absorption [82] (Fig. 2e), which are noted as the intrinsic loss of the microresonator. Consequently, the efficiency of emission into the waveguide is inherently limited by the loading factor $\eta$. Increasing $\eta$ up to unity is possible through strong over-coupling of the microresonator at the expense of a reduction in *Q*. The enhancement of comb-generating processes, on the other hand, scales with *Q* for EO modulation and $Q^2$ for FWM, thereby creating a trade-off between emission efficiency and total power consumption, which can be reconciled by improving the intrinsic *Q* of the devices.

## Microcombs generated in coupled-microresonators

**Table:** 

Box 2: Formalism of coupled resonators and general critical coupling	
*Two-level system*: Consider a simple case where two modes (frequencies: $\omega _{1,2}$; loss rates: $\kappa _{1,2}$) are coupled at a rate of *g*. The new eigenfrequencies of the system can be obtained by diagonalizing the following 2$\times$2 matrix:	
$$\begin{bmatrix}\omega _1+i\kappa _1/2 &{} g \\ g &{} \omega _2+i\kappa _2/2 \end{bmatrix},\qquad \qquad \qquad (6)$$	
which is given by	
$$\omega _\pm =\frac{\omega _1+\omega _2}{2}+i\frac{\kappa _1+\kappa _2}{4} \pm \frac{\sqrt{[\omega _1-\omega _2+i(\kappa _1-\kappa _2)/2]^2+4g^2}}{2}.\qquad \qquad \qquad (7)$$	
If *g* exceeds the differences in loss rates of the two modes, the hybridized modes exhibit a larger frequency splitting than the uncoupled modes, a phenomenon known as avoided-mode-crossing. Alternatively, the linewidths are altered in cases where *g* is smaller.	
*General critical coupling*: The concept of critical coupling extends to systems with multiple modes and loss channels. In the case of a two-mode problem, the general critical coupling can be derived from the following input-output relation [91]:	
$$\frac{\text{d}a_1}{\text{d}t}=-(i\Delta _1 + \kappa _1/2)a_1 -\text{i} g a_2 + \sqrt{\kappa _\text{e1}P_\text{in}},\qquad \qquad \qquad (8)$$	
$$\frac{\text{d}a_2}{\text{d}t}=-(i\Delta _2 + \kappa _2/2)a_2 -\text{i} g a_1\qquad \qquad \qquad (9)$$	
In this context, mode $a_1$ represents the pump mode, while mode $a_2$ is coupled to $a_1$ with a coupling rate *g*. Mode $a_2$ can correspond to any frequency mode, such as an additional mode introduced by a second cavity or a mode exhibiting a different spatial profile, polarization, or propagation direction within the same cavity. The formation of OFCs introduces nonlinear loss, which is accounted for in the total damping rates ($\kappa _1$ and $\kappa _2$). By substituting the steady-state solution of $a_2$ into Eq. (8), we obtain:	
$$\frac{\text{d}a_1}{\text{d}t}=-i\left( \Delta _1-\frac{g^2\Delta _2}{\Delta _2^2+\kappa _2^2/4}\right) a_1 -\left( \frac{\kappa _1}{2}+\frac{g^2\kappa _2/2}{\Delta _2^2+\kappa _2^2/4}\right) a_1 + \sqrt{\kappa _\text{e1}P_\text{in}}.\qquad \qquad \qquad (10)$$	
This equation provides the general critical coupling condition for mode $a_1$ from the loading perspective as	
$$\eta =\frac{\kappa _{\text{e1}}}{\kappa _1 + \frac{g^2\kappa _2}{\Delta _2^2 + \kappa _2^2/4}}=\frac{1}{2}.\qquad \qquad \qquad (11)$$	
It is worth noting that the mode hybridization introduces an additional detuning term $-\frac{g^2\Delta _2}{\Delta _2^2+\kappa _2^2/4}$, which can compensate for the pump detuning $\Delta _1$. An example of this is when $a_1$ serves as the pump mode for bright soliton microcombs.	

**Fig. 4:**
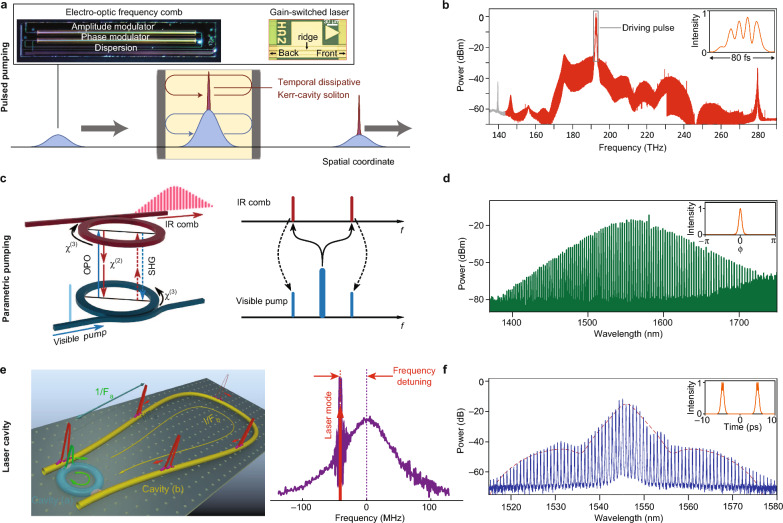
High-efficiency microcombs generated by increasing the pumping bandwidth.** a** Schematic configuration [92] of pulsed-pumped microcombs. The driving pulses can be derived from either an EO comb [20, 93] or a gain-switched laser [94]. **b** Optical spectrum of pulsed pumped microcomb [95]. Inset: intracavity intensity profiles of microcomb. **c** Parametrically pumped soliton microcombs [62]. **d** Optical spectrum of parametrically pumped microcomb [62]. Inset: intracavity intensity profiles of microcomb. **e** Laser cavity soliton microcombs [96, 97]. **f** Optical spectrum of laser cavity soliton microcomb [96]. Inset: intracavity intensity profiles of microcomb. Figures reproduced with permission from: **a** schematic configuration, ref. [92], Copyright 2017 Springer Nature; **a** image of the EO comb, ref. [93], Copyright 2022 Springer Nature; **a** image of the gain-switched laser, ref. [94], Copyright 2021 Springer Nature; **b** ref. [95], Copyright 2022 Springer Nature; **c d** ref. [62], Copyright 2021 Springer Nature; **e** left panel, ref. [96], Copyright 2019 Springer Nature; **e** right panel, ref. [97], Copyright 2022 Springer Nature. textbff ref. [96], Copyright 2019 Springer Nature

Recently, flourishing research interest has been steered toward generating microcombs in coupled resonators, due to the underlying complex nonlinear dynamics [88, 98, 99] and versatile capability for dispersion engineering [61, 100–102]. These devices consist of two or more microresonators in which optical modes couple to form hybridized resonant structures with reconfigurable energy levels and linewidths [91, 103–105]. Pumping a hybridized mode results in distinct input–output relations, which could improve the injection efficiency of microcombs and bypass the aforementioned theoretical limits (see Box 2). The concept of general critical coupling (GCC), which was originally proposed in Ref. [91], provides a guideline for maximizing the energy flow into the main microresonator (the resonator responsible for comb generation). Figure 3 presents several promising configurations based on GCC for realizing high-efficiency microcombs.

*GCC in Kerr microcombs.* For bright solitons, the far red-detuned pump has prohibited the realization of critical coupling. One method to realize GCC is by introducing a designated avoided-mode-crossing to the pump mode to compensate for the red-detuning requirement. In Ref. [84], the avoided-mode-crossing is achieved by evanescently coupling an auxiliary microresonator to the main comb-forming microresonator (Fig. 3a). The lower-frequency hybridized mode is made resonant with the pump by controlling the frequencies of uncoupled modes with thermo-optics. Remarkably, a 51% conversion efficiency for a single-soliton microcomb is demonstrated, showing a 10-fold improvement compared with single-soliton microcombs generated in standalone microresonators. Selective avoided-mode-crossings can also be introduced by periodically modulating the periphery of the microresonator [86, 106–111] (Fig. 3e). These gratings create a one-dimensional photonic crystal microresonator, in which degenerate counter-propagating modes are coupled. The coupling of modes with the same azimuthal number as the number of grating elements is enhanced by Bragg scattering, resulting in a fixed frequency splitting related to the sizes of the grating elements. In these devices, both bright solitons [86] and dark pulses [108] have been generated, displaying broadband spectra and conversion efficiency of up to 21%.

A technically similar yet fundamentally different approach to realizing GCC in Kerr microcombs was proposed in Ref. [85]. Here the pump enters the auxiliary microresonator before reaching the main microresonator. The resulting comb is then collected at the drop port (Fig. 3b). Ideally, the pump should recirculate within the auxiliary microresonator until it is entirely consumed by the comb generation processes in the main microresonator. To achieve this, the auxiliary microresonator needs to be strongly over-coupled to the input bus waveguide, exhibiting a significantly broader linewidth compared to the main microresonator. Experimental tests conducted on coupled fiber-ring resonators demonstrated an efficiency of 0.0046%, attributed to the small duty cycles of solitons within these kilometer-long resonators, which is expected to reach near unity in microresonators. Remarkable improvements in efficiency were observed at the through port, where over 99% of the pump power was consumed through the recycling scheme. Additionally, another approach to achieve power recycling is through the incorporation of a Mach-Zehnder-type bus waveguide, which couples to the microresonator multiple times [112]. By enhancing coupling at the pump wavelength, a 55% conversion efficiency is made possible in the case of soliton crystals.

*GCC in resonant EO microcombs.* The resonant EO microcombs serve as another example of GCC, offering a relatively straightforward mechanism in contrast to Kerr microcombs. In a single-resonator EO microcomb, the resonator is typically strongly under-coupled when driven by a powerful microwave tone. To compensate for the loss induced by the microwave, one can increase the external coupling between the cavity and waveguide, resulting in critical coupling for the EO microcomb. However, this approach significantly reduces the *Q* of the resonator, thus limiting the span of the EO microcomb. To overcome this limitation, a strongly over-coupled auxiliary resonator is employed to couple the pump power into the main resonator (Fig. 3c). This ensures that only the pump mode is over-coupled while all other modes remain under-coupled. By establishing a GCC on the auxiliary resonator, the pump efficiently flows into the main cavity for comb generation, preventing circulation within the auxiliary cavity. Resonant EO microcombs based on GCC [20] have been demonstrated on thin-film lithium niobate with a conversion efficiency 100 times higher (30%) and a span 2.2 times wider (132 nm) than the previously acclaimed state-of-the-art resonant EO microcombs generated in single microresonators [19].

## Broadband-pumped microcombs

Microcombs pumped by c.w. lasers typically manifest as pulses superimposed on a homogeneous background within the microresonator. While this background minimally contributes to the power of the effective comb lines, it consumes a significant amount of pump power. However, microcombs are also compatible with various alternative pump schemes, which do not conform to the theoretical efficiency limits derived for c.w. laser pumping. An overview of these different pump schemes is provided in Fig. 4.

*Pulse-pumped microcombs*. Synchronous pumping has been a widely adopted method for generating ultrashort pulses in optical parametric oscillators, where a pulsed pump co-circulates with the pulses formed in the resonator. This concept was introduced to Kerr microcombs in the form of picosecond-pulse-pumped solitons in a fiber-based Fabry-P$\acute{\text{e}}$rot microresonator by Herr et al. [92] (Fig. 4a). The pump pulse is derived from an EO frequency comb that comprises several modulators and a dispersion compensating unit, providing a widely tunable repetition frequency and carrier offset frequency [113]. Other feasible sources, such as resonant EO microcombs and gain-switched lasers, have also been explored [94]. The improved temporal overlap between the pump and the soliton enhances the efficiency by a factor inversely proportional to the duty cycle of the driving pulse train. Soliton operation allows for a small offset between the repetition frequencies of the pump and the FSR of the microresonator; however, the efficiency decreases for larger offsets [114]. Reference [115] derived the maximum efficiency as

12 $$\begin{aligned} \Gamma _\text{pulse}=2\pi ^2\eta ^2\tau /\tau _\text{p}, \end{aligned}$$

where $\tau$ and $\tau _\text{p}$ represent the duration of the soliton and the pump pulse. For sinc-shaped pump pulses, $\Gamma _\text{pulse}$ is $N_\text{p}$ times larger than $\Gamma _\text{BS}$, where $N_\text{p}$ is the number of spectral lines in the pump. The maximum efficiency achieved is 54% in a strongly over-coupled silica disk microresonator [115]. We note that, for pulse-pumped microcombs, the overall conversion efficiency should include the conversion efficiency of the pulsed pump generation itself, which is typically not included in the reported efficiency. For example, a non-resonant EO comb typically costs several-watt microwave power, along with considerable optical insertion loss ($>10$ dB), to synthesize sub-picosecond pulses.

**Fig. 5:**
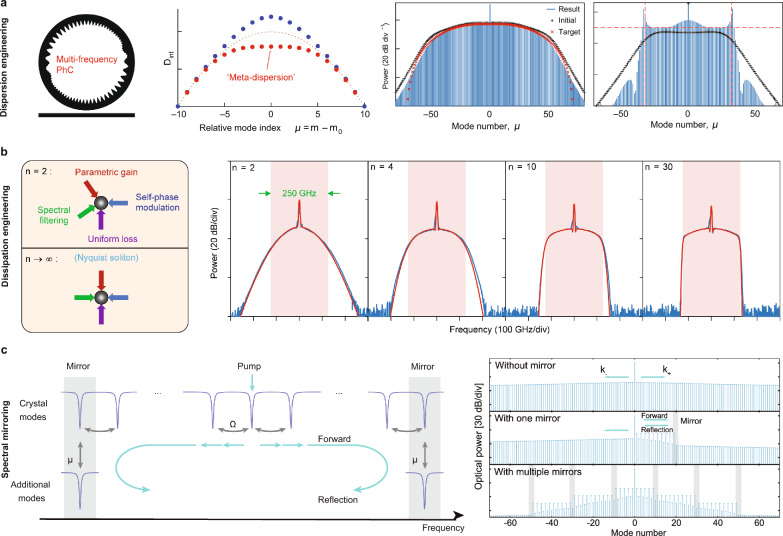
Flat-top microcombs.** a** Generation of Kerr microcombs in multi-frequency photonic crystal microresonators by creating meta-dispersion via Bragg scattering [116]. **b** Generation of Nyquist soliton by introducing spectral filtering within a Kerr resonator [117]. **c** Generation of flat-top resonant EO microcombs by introducing avoided-mode-crossings (“mirrors”) in the frequency domain [118]. Figures reproduced with permission from: **a** ref. [116], Copyright 2023 Springer Nature; **b** ref. [117], Copyright 2023 Springer Nature; **c** ref. [118], Copyright 2022 Springer Nature

Pulsed pumping has important applications in realizing broadband microcombs at electronically-detectable repetition frequencies. Anderson et al. [95] generated a nearly octave-spanning microcomb with 28 GHz $f_r$ in a Si$_3$N$_4$ microresonator by pulsed pumping at the wavelength where the group velocity dispersion is close to zero, along with the formation of higher-order dispersive waves (Fig. 4b). Instead of a standalone pulse, the intracavity waveform appears to have multiple peaks due to the co-existence of normal and anomalous dispersion within the span of the microcomb. Recently, Xiao et al. [119] achieved a span of 2/3 octave in a pulsed-pumped highly-nonlinear Fabry-P$\acute{\text{e}}$rot microresonator with a 10-GHz-rate soliton microcomb. These microcombs provide over 5,000 comb lines, which have direct applications in astronomy for the broadband calibration of spectrometers [39].

*Parametrically-pumped microcombs*. The potential to pump dissipative Kerr solitons at twice their carrier frequency through parametric down-conversion has been proposed since the early 1990 s [120–123]. The parametrically driving scheme requires the resonator to support both $\chi ^{(2)}$ and $\chi ^{(3)}$ nonlinearity simultaneously (Fig. 4c). While the bandwidth of the $\chi ^{(2)}$ parametric gain is narrower than the four-wave mixing process, it can be extended by periodic poling. Recent experimental demonstrations have been achieved in fiber resonators [124] and AlN microresonators [62], using the pump wavelength in the visible range and generating solitons in the infrared (IR). In AlN microresonators, multiple pairs of comb lines gain from the parametric down-conversion, resulting in a remarkable efficiency of 17%, which is more than one order of magnitude higher than the c.w. pumping method. The intracavity waveform for the IR soliton is also found to be free of background (Fig. 4d). Furthermore, the $\chi ^{(2)}$ process in these doublet-resonant microresonators creates an effective Kerr nonlinearity [125], offering the promise of producing pure Pockels solitons.

*Laser-cavity soliton microcombs*. In contrast to the parametric process, active media such as rare-earth ions can store unused energy for later use. Embedding a microresonator within a laser cavity enables lasing at the resonances, removing the need for a narrow-linewidth pump laser (Fig. 4e) [96]. The laser frequency is determined by modified round-trip phase relations and can be slightly adjusted around the resonances of the microresonator by tuning the length of the external cavity. The red-detuning of the pump frequency leads to the formation of solitons in the microresonator, fulfilling a simple criterion for self-starting the mode-locking process (Fig. 4f) [97]. The energy structure of the Er$^{3+}$ ions provides considerable gain for comb lines across telecommunication C-band, such that the center of the spectrum is notably stronger than other parts, and the envelope deviates from the sech$^2$ shape (Fig. 4g). The waveform is also free from a continuous-wave background since the Er$^{3+}$ ions only amplify the circulating soliton pulses. The mode efficiency, which describes the energy ratio of the comb without the strongest comb line, is reported to be as high as 75% in this configuration [96, 126]. A more strict definition of efficiency should refer to the optical power used to pump the Er$^{3+}$ ion.

## Flat-top microcombs

The flatness of the microcomb spectra is another vital parameter as modern communication systems have strict standards for the power emitted in each channel. Although the cascaded parametric processes could broaden the spectra of microcombs, the majority of the created comb lines are notably weaker than those near the center of the spectra due to group velocity dispersion. Indeed, the performances of many applications based on wavelength division multiplexing technology are often limited by the weakest comb lines. Several strategies have been proposed to realize flat-top microcombs by modifying the dispersion and loss of the resonant structures.

*Dispersion engineering*. The dispersion is the most crucial factor that determines the spectral shape of the microcomb [127]. While the sech$^2$-shaped bright soliton arises solely from second-order anomalous dispersion, in mode-locked lasers a flatter spectrum could appear if higher-order dispersion dominates [128]. Li et al. demonstrated that the local dispersion within a microring can be adjusted from anomalous to normal by varying the waveguide width [129]. This dispersion-managed microresonator effectively eliminates second-order dispersion and instead relies on higher-order dispersion, resulting in a Gaussian-shaped soliton microcomb with a more evenly distributed spectrum centered around the pump wavelength. Another approach to controlling dispersion involves multi-frequency photonic crystal microresonators, which leverage multiple split-resonances to achieve a “meta-dispersion” effect (Fig. 5a) [107, 116, 130]. In this configuration, Kerr microcombs produced in a set of resonances exhibit distinct spectra due to the flexible dispersion profile. By incorporating inverse-design algorithms to solve the Lugiato-Lefever equation, it becomes possible to tailor the comb spectra according to specific requirements, such as desired span, power, and spectral flatness.

*Dissipation engineering*. Flat-top microcombs can also be achieved through the termination of the cascaded nonlinear process. In a recent study by Xue et al. [117], it was demonstrated that the spectrum of a Kerr comb can be reshaped by incorporating additional spectral filtering in a fiber-ring resonator (Fig. 5b). The comb lines outside the desired frequency range are suppressed with increased filtering strength, and the comb power is more evenly distributed within this range. Proper dispersion gives a Nyquist microcomb which has uniform comb power across its spectrum. However, implementing spectral filtering in typical ring-shaped or whispering-gallery-mode microresonators can be challenging. In contrast, Fabry-P$\acute{\text{e}}$rot microresonators naturally possess the requisite spectral filtering capabilities. The viability of generating soliton microcombs in integrated Fabry-P$\acute{\text{e}}$rot microresonators has been demonstrated [131].

*Spectral mirroring*. Unlike the Kerr effect, which facilitates broadband parametric gain, resonant EO microcombs exhibit coupling between individual comb lines primarily with their nearest neighbors. The introduction of avoided-mode-crossings – disruptions that abruptly reshape the resonant frequencies of selected modes – serves to terminate the cascading process and redirect the energy flow [118]. Figure 5c illustrates a mirror-like effect in the frequency domain, exhibiting periodic fringes reminiscent of a standing wave. Notably, the spectrum exhibits flattening at alternating lines due to interference effects. This mirror-induced reflection traps optical energy within the microresonator, preventing its distribution across the frequency spectrum and thus amplifying the energy between the two frequency mirrors. This phenomenon enables spectral engineering in EO microcombs. While the pump power is significantly higher than the comb lines, it can be effectively managed using the GCC on EO microcomb generators [20]. Comparable boundary effects have also been demonstrated in fiber-cavity systems [132]. The practical implementation of avoided-mode-crossings in these devices involves either counterpropagating modes [133], distinct transverse modes, and coupled microresonators [118].

## Summary and outlook

Beyond the conversion efficiency discussed in this review, in practical applications the wall plug efficiency (WPE) of the laser diodes, optical amplifiers, and RF drivers used to pump the microcomb must be quantified to provide a critical assessment of the total electrical power consumption. By using laser diodes (WPE>20%) to pump high-efficiency microcombs, it is possible to achieve WPE similar to those of state-of-the-art semiconductor mode-locked lasers (WPE$\sim$15%) [134, 135]. To this end, the interface loss between laser diodes and high-*Q* microresonators should also be minimized through heterogeneous integration [59, 136].

High-efficiency microcombs could have revolutionary impacts across various fields. For telecommunications, near-unity microcomb efficiency could yield electrical-optical power conversion efficiencies equivalent to laser arrays. Once the efficiency is improved to near-unity, the next frontier is increasing the power per comb line while maintaining a high overall WPE, which ensures substantial optical signal-to-noise ratios even after amplification. Moreover, the well-defined channel spacings of microcombs would eliminate the need for complex temperature control systems typically required for individual lasers, significantly reducing total power consumption. As a result, we anticipate the integration of microcombs into optical modules that provide multiple channels of wavelength access within the next decade. This expectation is supported by the demonstrated capabilities of petabit-per-second communications [43] and compatibility with silicon photonics [49, 137].

Extending the spectral reach of microcombs beyond the near-infrared bands holds great potential for enabling diverse spectroscopic applications. Particularly, visible-wavelength combs play a pivotal role in the hunt for exoplanets, as they facilitate the calibration of Doppler shifts in the spectra of solar-like stars [138]. On the other hand, molecular fingerprinting primarily focuses on the mid-infrared spectral window [139]. However, the integrated laser technologies at visible and mid-IR wavelengths are still evolving and currently offer lower power compared to those in telecommunication bands. Therefore, high-efficiency microcombs are crucial to address the need for high-power pump lasers. By mitigating the power requirements, we could facilitate the realization of broadband optical frequency combs at these wavelengths, providing compact spectroscopic tools for a wide range of interdisciplinary research [140–144].

Currently, microwave-to-optical links relying on spectrally-broadened microcombs [145] or multiple phase-locked microcombs are quite sophisticated [37, 40, 146]. This highlights the need for octave-spanning microcombs that can be easily self-referenced, simplifying the system and ensuring the detection of the carrier-envelope-offset frequency. Such devices would generate approximately 10,000 comb lines, and the comb lines used for self-referencing must possess sufficient power for a reliable signal-to-noise ratio of the carrier-envelope-offset frequency. We note that the design of microresonators should be carefully optimized to support broadband microcombs, considering many effects often overlooked in narrowband microcombs. For instance, using higher-order dispersion to compensate for Raman effects could extend the potential bandwidth of microcombs [16, 147]. Besides, the reported high efficiencies in existing literature are achieved under optimized operating conditions, warranting further investigation into their applicability across wider spectral ranges. The ultimate solution may involve implementing a combination of protocols for high-efficiency microcombs, including pumping coupled resonators in a parametric or pulsed manner.

Microcombs continue to evolve, improving their efficiency, coherence, ease of operation, and other key aspects, gradually approaching the level of existing table-top OFCs. At present, most OFCs are only available in research laboratories. However, within the next decade, this valuable technology should become as accessible as semiconductor lasers in terms of size, weight, power consumption, and cost. We eagerly anticipate the long-lasting impact of microcomb technology, including its potential to speed up internet communications, improve navigation precision, and test fundamental physics.

## Data Availability

Not applicable.
